# Integrated Plasma snoRNA–Protein Network Analysis Identifies Circulating Molecular Signatures Associated with High-Risk Benign Breast Lesions and Early Breast Cancer

**DOI:** 10.3390/ijms27219530

**Published:** 2026-10-25

**Authors:** Mayumi Jijiwa, Vedbar S. Khadka, Matthew Huo, Lauren Higa, Shaina Young, Youping Deng

**Affiliations:** 1 Department of Quantitative Health Sciences, John A. Burns School of Medicine, University of Hawaii, Honolulu, HI 96813, USA; jijiwa@hawaii.edu (M.J.); vedbar@hawaii.edu (V.S.K.); higalaur@hawaii.edu (L.H.); sfy@hawaii.edu (S.Y.); 2 Krieger School of Arts and Sciences, Johns Hopkins University, Baltimore, MD 21218, USA; mhuo3@jh.edu

**Keywords:** high-risk benign breast lesion, early breast cancer, snoRNA, liquid biopsy, proteomics, multi-omics, co-expression network, ECM, platelet

## Abstract

High-risk benign breast lesions increase subsequent breast cancer risk, yet circulating molecular patterns that distinguish them from early breast cancer remain poorly defined. We profiled plasma small nucleolar RNAs (snoRNAs) and small Cajal body-specific RNAs (scaRNAs) using UMI-based small RNA sequencing in 32 plasma samples from patients with low-risk benign lesions (Be; 9), high-risk benign lesions (HB; 14), or early breast cancer (CA; 9), and integrated matched data-independent acquisition (DIA) proteomics in a subset of 20 samples (HB; 11, CA; 9). Weighted gene co-expression network analysis (WGCNA) of 475 molecular features identified 11 modules. The Yellow module contained 25 snoRNAs/scaRNAs and 26 proteins and was enriched for extracellular matrix (ECM), blood microparticle, platelet, and serine-type endopeptidase inhibitor functions. An exploratory Yellow-module-focused analysis identified 10 snoRNAs with nominally lower abundance in CA than in HB (*p* < 0.05), showing concordant directions of differential abundance with the global analysis. The strength of differential RNA evidence correlated with mean intramodular RNA–protein correlation (Spearman ρ = 0.788, *p* = 2.85 × 10^−6^). External snoDB target mapping supported 28.6% of the exploratory network edges, including SNORD38A–FN1, SNORA73B–LAMB1, SNORD22–LAMB1, and SNORD22–NCAM1. Integration of differential plasma snoRNA profiling, cross-omic network analysis, and experimentally observed snoRNA interaction data indicated convergence on ECM/collagen and platelet/coagulation biology. These candidate snoRNA–protein axes may characterize differences in the circulating molecular states of CA and HB.

## 1. Introduction

Breast cancer remains a leading cause of cancer-related mortality among women worldwide [1]. Early detection and precise risk stratification are essential to balance timely intervention with the avoidance of over-diagnosis and over-treatment. Clinically, benign breast lesions represent a highly heterogeneous spectrum. Non-proliferative lesions (such as fibroadenomas and simple cysts) carry little to no increased risk of subsequent breast cancer. Conversely, proliferative lesions with atypia, including atypical ductal hyperplasia (ADH), atypical lobular hyperplasia (ALH), and flat epithelial atypia (FEA), confer a substantial, long-term cumulative risk of malignant transformation [2,3,4,5,6,7,8,9,10,11]. Although histopathological examination of core needle biopsies remains the gold standard for diagnosis, tissue biopsy is invasive, subject to sampling bias, and provides limited insight into systemic biological alterations accompanying early disease progression. Minimally invasive blood-based biomarkers could therefore provide complementary biological information.

Liquid biopsies have emerged as promising tools for non-invasive disease monitoring [12]. While circulating tumor DNA (ctDNA) and circulating tumor cells (CTCs) have demonstrated utility in advanced malignancies, their abundance in early-stage breast cancer is frequently below detection limits [12,13,14]. Consequently, attention has expanded toward circulating non-coding RNAs and plasma proteins. Plasma reflects not only tumor-derived factors but also broader host-systemic responses, including extracellular matrix (ECM) remodeling, local inflammation, tissue repair, and hemostatic/platelet reactivity [15,16]. Integrative approaches that evaluate coordinated behavior across molecular layers may therefore reveal disease-associated structure that is not evident from single-analyte testing.

Small nucleolar RNAs (snoRNAs) are 60–300-nucleotide non-coding RNAs primarily recognized for guiding canonical post-transcriptional modifications of ribosomal RNAs, including 2′-O-methylation by C/D box snoRNAs and pseudouridylation by H/ACA box snoRNAs [17,18,19,20]. Small Cajal body-specific RNAs (scaRNAs), a related class of small non-coding RNAs localized to Cajal bodies, guide analogous modifications of spliceosomal small nuclear RNAs [21,22]. Emerging evidence indicates that snoRNAs also regulate alternative splicing, translational efficiency, metabolic stress responses, and oncogenic signaling pathways [19,22,23,24,25]. Because snoRNAs are highly stable in blood plasma, encapsulated within extracellular vesicles or bound to protective ribonucleoprotein complexes, they represent attractive liquid biopsy candidates [26,27,28]. However, whether circulating snoRNA dynamics distinguish high-risk benign lesions from early invasive malignancy remains unexplored. Furthermore, single-analyte biomarker strategies often fail in early disease stages due to high inter-individual variation. Integrative multi-omic approaches that capture modular, highly correlated networks across molecular layers offer enhanced sensitivity to detect subtle biological shifts.

In this exploratory study, we profiled plasma snoRNAs/scaRNAs across three clinical groups: benign lesions without established increased risk (Be), high-risk benign lesions (HB), and early breast cancer (CA). We then generated plasma proteomic data using data-independent acquisition (DIA) mass spectrometry in a paired CA–HB subset. Across these two omics layers, we first assessed global profiles and group-level differences in individual molecular features, then constructed an integrated weighted correlation network to examine whether differential RNA findings converged on cross-omic modules. Finally, we used experimentally observed RNA interactions catalogued in snoDB to provide external functional context for candidate cross-omic associations. Rather than developing a diagnostic classifier, we aimed to identify coordinated circulating molecular patterns and candidate snoRNA–protein axes that may characterize biological differences between HB and CA and warrant future validation.

## 2. Results

### 2.1. Clinicopathological Characteristics of the Plasma Samples

The study included 32 plasma samples from White women: 9 with benign breast lesions without an established increase in breast cancer risk (Be), 14 with high-risk benign breast lesions (HB), and 9 with early breast cancer (CA) (Table 1 and Table S1). Median age at plasma collection was 54 years (interquartile range [IQR], 50–63) in Be, 51 years (IQR, 49–64) in HB, and 61 years (IQR, 47–64) in CA. Median body mass index (BMI) was 26, 25, and 30 kg/m^2^, respectively. Smoking history varied across the groups, and the CA group included two current smokers.

The HB group consisted primarily of ADH, ALH, FEA, and lesions containing combinations of these features. The Be group included fibroadenoma and fibrocystic changes. The CA group comprised eight early-stage invasive cancers and one ductal carcinoma in situ (DCIS), predominantly pT1N0, and included both luminal A and triple-negative tumors. A subset of 20 samples (9 CA and 11 HB) with matched RNA and proteomic data was used for multi-omic network integration (Section 2.4). Given the sample size and clinical heterogeneity, all subsequent analyses were considered exploratory.

### 2.2. Plasma snoRNA Profiles

**Figure 1:**
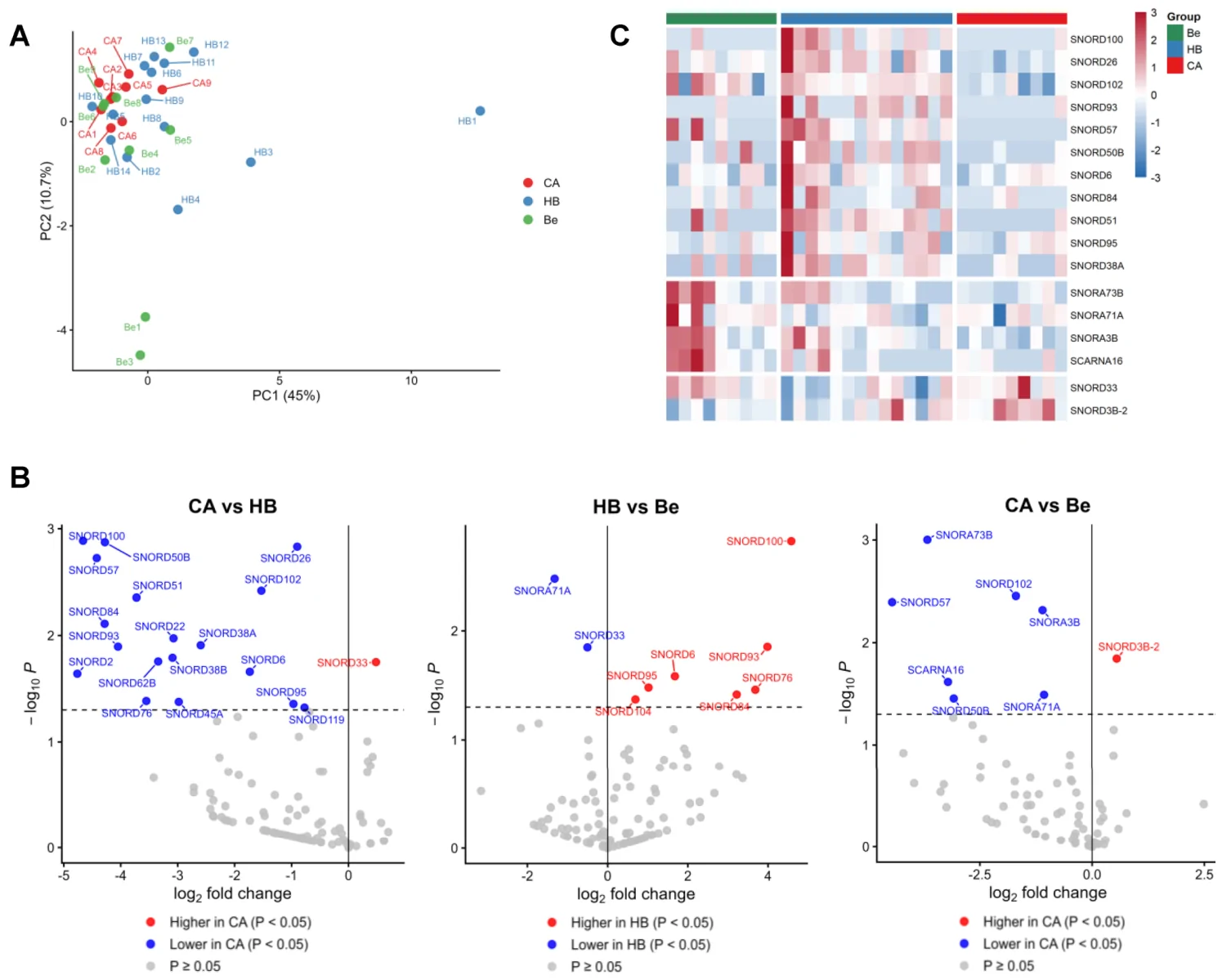
Plasma snoRNA profiles across benign breast lesions and early breast cancer. (**A**) Principal component analysis of variance-stabilizing transformed UMI counts for 149 plasma snoRNA/scaRNA features from patients with early breast cancer (CA, n = 9), high-risk benign breast lesions (HB, n = 14), and benign lesions without established increased cancer risk (Be, n = 9). Percentages indicate the variance explained by each principal component. (**B**) Volcano plots of differential snoRNA/scaRNA expression for CA vs. HB, HB vs. Be, and CA vs. Be. Red and blue indicate snoRNA/scaRNA features with nominal *p* < 0.05 and higher or lower expression, respectively, in the first group of each comparison; gray indicates *p* ≥ 0.05. The dashed line indicates *p* = 0.05. (**C**) Heatmap of 17 snoRNAs/scaRNAs with nominal *p* < 0.05 by DESeq2 LRT across Be, HB, and CA. Expression values were variance-stabilized and standardized to row Z-scores for each RNA across samples.

GeneGlobe analysis detected 159 features in the other RNA category (Supplementary Material S1). After nomenclature harmonization and definition of the analysis set, 149 small non-coding RNA features, including 136 snoRNAs and 13 scaRNAs, were used for principal component analysis of all 32 samples (Figure 1A). Principal components 1 and 2 explained 45.0% and 10.7% of the total variance, respectively. The three clinical groups overlapped substantially, and the global plasma RNA profile did not clearly separate Be, HB, and CA. Several Be and HB samples were displaced in principal-component space, but no consistent group-specific cluster was observed.

Pairwise differential-expression analyses were performed for CA vs. HB, HB vs. Be, and CA vs. Be within the 149 snoRNA/scaRNA cohort (Figure 1B). The volcano plots highlight features with nominal *p* < 0.05. Nominally different features in the CA vs. HB comparison were predominantly lower in CA, whereas the other two comparisons showed more heterogeneous directional patterns.

To assess expression differences across all three clinical groups simultaneously, a DESeq2 likelihood ratio test (LRT) was performed. Seventeen snoRNAs/scaRNAs had nominal *p* < 0.05, although none remained significant at FDR < 0.05 (Figure 1C). Eleven showed relatively higher mean abundance in HB, whereas four—SNORA73B, SNORA71A, SNORA3B, and SCARNA16—showed relatively higher mean abundance in Be. SNORD33 and SNORD3B-2 exhibited distinct patterns that did not conform to either predominant profile. Overall, the heatmap revealed heterogeneous group-associated patterns rather than a uniform directional change across Be, HB, and CA.

### 2.3. Plasma Proteomic Profiles

**Figure 2:**
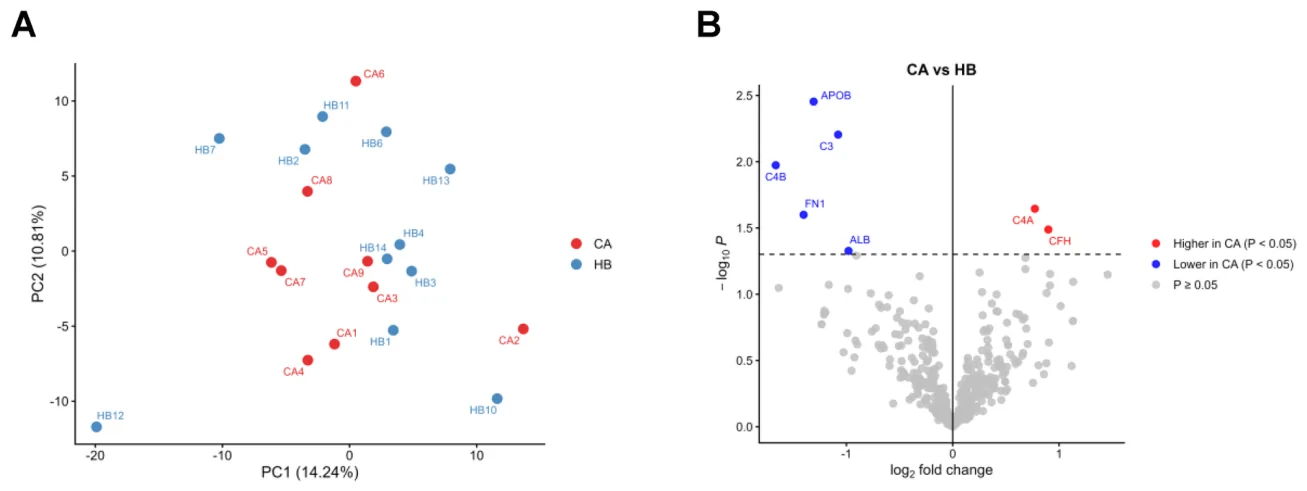
Plasma protein profiles in high-risk benign breast lesions and early breast cancer. (**A**) Principal component analysis of 394 proteins with complete measurements from participants with early breast cancer (CA, n = 9) and high-risk benign breast lesions (HB, n = 11). Log_2_ cyclic-loess-normalized protein intensities were used. Percentages indicate the variance explained by each principal component. (**B**) Volcano plot showing differential abundance of all 422 detected proteins between CA and HB. Red and blue indicate proteins with nominal *p* < 0.05 and higher or lower abundance, respectively, in CA; gray indicates *p* ≥ 0.05. The dashed line indicates *p* = 0.05.

Quantitative proteomic profiling was performed on the CA–HB subset with matched RNA and proteomic data, including 9 CA and 11 HB samples (Table S1). Data-independent acquisition mass spectrometry detected 422 plasma proteins (Supplementary Material S2). Principal component analysis using the 394 proteins with complete measurements across all 20 samples showed substantial overlap between CA and HB; principal components 1 and 2 explained 14.24% and 10.81% of the variance, respectively (Figure 2A).

Differential-abundance analysis of all 422 proteins identified seven proteins with nominal *p* < 0.05: apolipoprotein B (APOB), complement component 3 (C3), complement C4-B (C4B), complement C4-A (C4A), fibronectin 1 (FN1), complement factor H (CFH), and albumin (ALB) (Figure 2B). APOB, C3, C4B, FN1, and ALB were lower in CA, whereas C4A and CFH were higher. None remained significant after Benjamini–Hochberg correction (FDR < 0.05). Thus, individual protein differences between CA and HB were modest, prompting investigation of coordinated cross-omic patterns through co-expression modeling.

### 2.4. Integrated snoRNA–Protein Co-Expression Network

**Figure 3:**
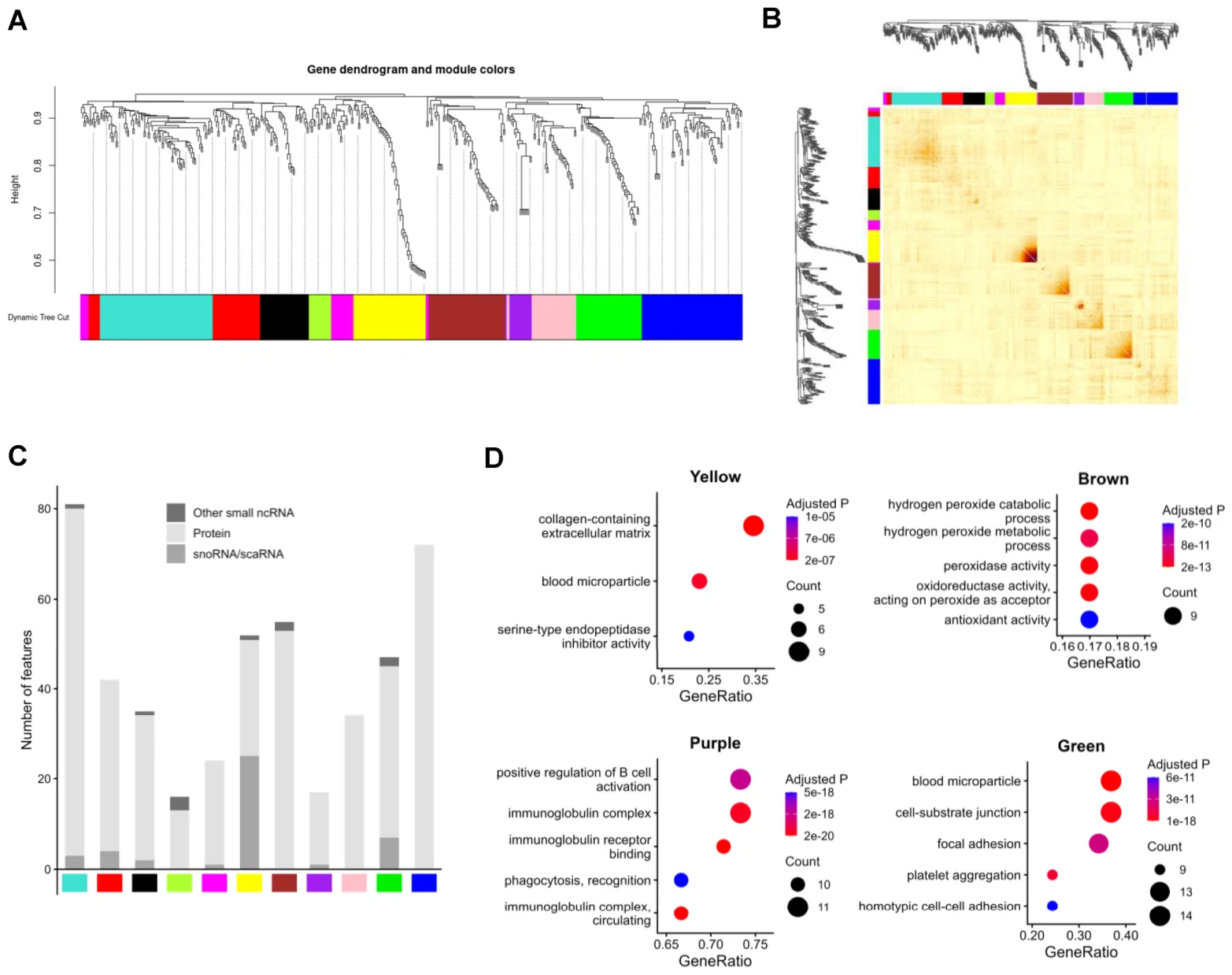
Integrated snoRNA–protein co-expression network. WGCNA was performed using 475 molecular features, comprising 422 proteins and 53 small non-coding RNAs, measured in 20 plasma samples with paired RNA and proteomic data. (**A**) Hierarchical clustering dendrogram and assignment of features to 11 co-expression modules. (**B**) Topological overlap matrix heatmap, with module assignments indicated by colored bars. (**C**) Numbers of snoRNA/scaRNA, other small non-coding RNA, and protein features in each module. (**D**) Gene Ontology over-representation analysis of the protein-coding genes corresponding to the protein components of the Yellow, Brown, Purple, and Green modules. Representative enriched terms are shown for each module. Dot size indicates gene count, and color indicates the adjusted *p* value. scaRNA, small Cajal body-specific RNA; snoRNA, small nucleolar RNA; WGCNA, weighted gene co-expression network analysis.

An integrated weighted co-expression network was constructed using the same 20 samples with matched RNA and proteomic data. The network included 475 molecular features, comprising 422 proteins and a filtered set of 53 small non-coding RNAs (Supplementary Material S1). Hierarchical clustering and dynamic tree cutting identified 11 co-expression modules (Figure 3A). The topological overlap matrix (TOM) revealed blocks of relatively high within-module connectivity (Figure 3B). Module composition varied substantially: many modules were dominated by proteins, whereas several contained both small non-coding RNAs and proteins (Figure 3C).

Gene Ontology (GO) over-representation analysis was performed for the protein-coding genes corresponding to the proteins in each of the 11 modules. Results for the Yellow, Brown, Purple, and Green modules, which showed prominent within-module connectivity in the TOM, are presented in Figure 3D. The Brown module was enriched for hydrogen peroxide catabolic and metabolic processes, peroxidase activity, and antioxidant activity. The Purple module was enriched for the immunoglobulin complex, B-cell activation, and immune-receptor-related functions. The Green module was enriched for blood microparticles, cell–substrate junctions, focal adhesion, and platelet aggregation. The Yellow module was enriched for collagen-containing extracellular matrix, blood microparticles, and serine-type endopeptidase inhibitor activity. The molecular compositions of these four modules are shown in Table 2.

### 2.5. Differential snoRNA Signal Converges on the Yellow Cross-Omic Module

**Figure 4:**
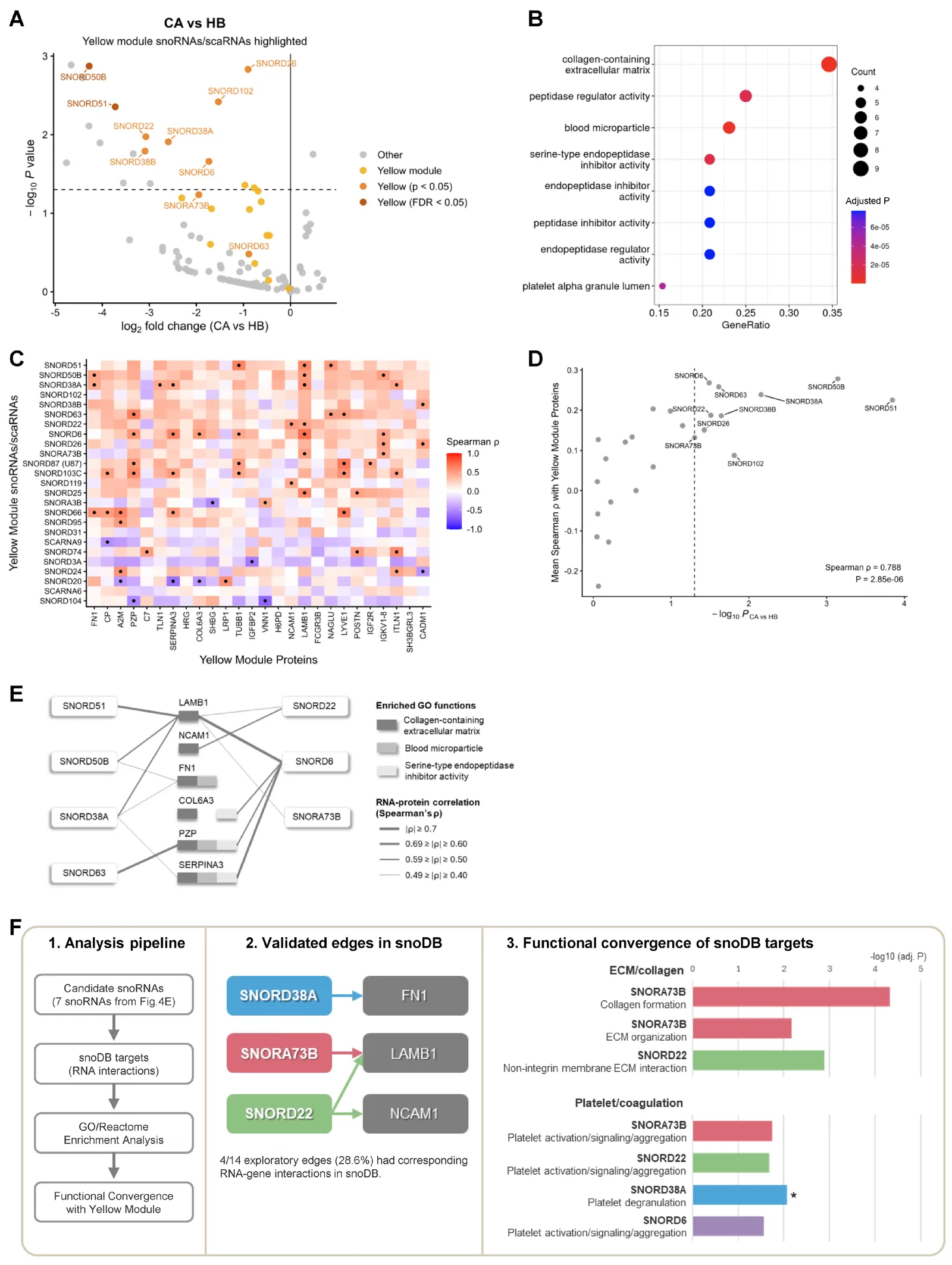
Characterization and external functional contextualization of the Yellow cross-omic module. (**A**) Yellow-module snoRNAs/scaRNAs overlaid on the global CA vs. HB differential-expression landscape. The volcano plot shows 149 snoRNAs/scaRNAs from the full cohort (9 CA vs. 14 HB; Figure 1B). Yellow-module RNAs are highlighted based on a separate analysis of 25 module RNAs in the matched cohort (9 CA vs. 11 HB): yellow, *p* ≥ 0.05; orange, *p* < 0.05 and FDR ≥ 0.05; brown, FDR < 0.05. FDR values were calculated within the 25 module RNAs and do not represent global discovery-level significance. (**B**) Gene Ontology gene set enrichment analysis of the 26 Yellow-module protein-coding genes ranked by fold change between CA and HB. Dot size indicates gene count, and color indicates the adjusted *p* value. (**C**) Spearman correlations between 25 Yellow-module snoRNAs/scaRNAs and 26 proteins across the 20 paired samples. Black dots indicate nominal *p* < 0.05. (**D**) Association between CA vs. HB differential-expression significance and the mean correlation of each snoRNA/scaRNA with Yellow-module proteins (Spearman ρ = 0.788, *p* = 2.85 × 10^−6^). (**E**) Exploratory network connecting seven CA–HB-associated snoRNAs, selected correlated proteins, and enriched functions. Edge thickness represents the absolute Spearman correlation coefficient. (**F**) External contextualization using experimentally observed snoDB interactions: (1) the target-mapping and enrichment workflow; (2) snoDB support for 4 of the 14 exploratory edges; and (3) representative ECM/collagen and platelet/coagulation pathways enriched among snoDB targets. Bar length represents −log_10_(adjusted *p*). The asterisk indicates SNORD38A platelet-degranulation enrichment exceeding all 100 size-matched random target sets (empirical *p* = 0.0099).

Notably, the Yellow module contained a relatively large number of snoRNAs/scaRNAs (Figure 3C and Table 2), many of which showed lower plasma abundance in CA than in HB in the differential-expression analysis (Figure 1B). We therefore focused on the Yellow module to determine whether its RNA components indeed differed in abundance between CA and HB. Among the 25 Yellow-module snoRNAs/scaRNAs, 10 showed nominal differential abundance in the 20-sample subset (*p* < 0.05), all with lower abundance in CA. In this exploratory module-restricted analysis, SNORD51 and SNORD50B had FDR < 0.05 after Benjamini–Hochberg correction within the 25 Yellow-module RNA features, although neither reached FDR < 0.05 in the global analysis of 149 snoRNAs/scaRNAs. Figure 4A maps the Yellow-module RNA features onto the global differential-expression landscape shown in Figure 1B (9 CA vs. 14 HB). This visualization illustrates the concordance in the direction of differential abundance between the Yellow-module-restricted and global CA vs. HB analyses. Thus, the differential RNA findings converged on a cross-omic module defined by unsupervised network analysis.

Gene Ontology gene set enrichment analysis (GSEA) of the 26 Yellow-module protein-coding genes ranked by CA vs. HB fold change highlighted collagen-containing extracellular matrix, blood microparticle and platelet-related terms, and peptidase or endopeptidase regulatory functions (Figure 4B). These findings were consistent with the functional themes identified by GO over-representation analysis of the Yellow module (Figure 3D).

Pairwise Spearman correlations were calculated between the 25 Yellow-module snoRNA/scaRNA profiles and the 26 protein profiles across the same 20 samples (Figure 4C). Sixty-two of the 650 RNA–protein pairs had nominal *p* < 0.05, although none remained significant after correction across all 650 tests. The pairwise correlations were therefore treated as exploratory. Across the 25 RNA features, stronger evidence of CA–HB differential abundance was associated with a more positive mean correlation with the 26 Yellow-module proteins (Spearman ρ = 0.788, *p* = 2.85 × 10^−6^; Figure 4D). This relationship supports coordinated cross-omic variation but does not distinguish within-group covariation from differences between group means.

An exploratory network retained 14 positive RNA–protein correlations with nominal *p* < 0.05, connecting seven CA–HB-associated snoRNAs with six Yellow-module proteins contributing to enriched functions (Figure 4E). The network linked SNORD51, SNORD50B, SNORD38A, SNORD63, SNORD22, SNORD6, and SNORA73B with LAMB1, NCAM1, FN1, COL6A3, PZP, and SERPINA3. Because none of the 650 correlations remained significant after multiple-testing correction, the network represents hypothesis-generating coordinated variation rather than evidence of direct regulation.

Experimentally observed RNA interactions for the seven network snoRNAs were subsequently retrieved from snoDB. Four of the 14 exploratory edges (28.6%) were supported by reported interactions between the snoRNA and transcripts encoding the corresponding protein: SNORD38A–FN1, SNORA73B–LAMB1, SNORD22–NCAM1, and SNORD22–LAMB1 (Figure 4F). Gene Ontology Biological Process and Reactome analyses of the experimentally observed protein-coding transcript targets indicated convergence on extracellular matrix/collagen processes for SNORA73B and SNORD22 and on platelet/coagulation processes involving SNORA73B, SNORD22, SNORD38A, and SNORD6. In a size-matched sensitivity analysis, platelet-degranulation enrichment for SNORD38A was stronger than that observed in all 100 random target sets of the same size (0/100; empirical *p* = 0.0099). Collectively, these analyses indicated convergence on extracellular matrix/collagen and platelet/coagulation biology across differential plasma RNA abundance, cross-omic network structure, and experimentally observed RNA-interaction data.

## 3. Discussion

There is a critical clinical need for non-invasive approaches to distinguish high-risk benign breast lesions from early invasive breast cancer. In this multi-omic study, we integrated plasma snoRNA/scaRNA sequencing with DIA proteomics to examine circulating molecular patterns across benign, high-risk benign, and early breast cancer groups. While global molecular landscapes exhibited broad overlap, multi-omic WGCNA uncovered a coordinated cross-omic module, the Yellow module, that links circulating snoRNAs with matrix remodeling, protease regulation, and platelet biology.

The substantial overlap among the three clinical groups in the PCA, despite PC1 explaining 45.0% of the total variance, suggests that the dominant variation in plasma snoRNA/scaRNA profiles may reflect interindividual biological heterogeneity and/or technical variation rather than disease status alone. Although pairwise differential-expression analysis identified 12 snoRNAs/scaRNAs with FDR < 0.05 in CA vs. HB, one in CA vs. Be, and none in HB vs. Be, these differences in a subset of RNAs may not have dominated the overall variation sufficiently to produce distinct clusters. The specific factors contributing to PC1 remain undetermined. Overall, pairwise and three-group analyses revealed heterogeneous group-associated patterns in circulating snoRNA abundance, predominantly characterized by higher abundance in HB than in CA. It is important to emphasize that lower abundance in plasma does not necessarily reflect intracellular downregulation within tumor tissues. Circulating non-coding RNAs may originate from diverse cellular compartments, including tumor cells, immune cells, vascular endothelial cells, and platelets, and may enter the circulation through extracellular vesicle secretion or passive release during tissue injury [16,28]. High-risk benign lesions, such as ADH and ALH, may be accompanied by stromal proliferation, localized inflammation, and tissue-repair responses that increase the release or retention of specific snoRNAs or their microparticle carriers. In contrast, early breast cancers may alter the cellular release, carrier distribution, stabilization, or clearance of circulating snoRNAs.

These findings argue against viewing benign, high-risk benign, and malignant lesions as a strict linear molecular continuum [2,3,4]. High-risk atypical lesions confer an increased future risk of breast cancer but do not necessarily progress to invasive disease, nor does a subsequent carcinoma necessarily arise directly from the sampled atypical lesion. High-risk benign lesions and early breast cancer may therefore represent distinct pathophysiological states associated with different systemic host responses.

The proteomic alterations observed in isolation were modest, with no single protein reaching FDR significance. This aligns with prior liquid biopsy studies in early-stage cancers, in which systemic protein signatures may be obscured by interindividual variability, BMI, and acute-phase inflammatory fluctuations [29]. However, by grouping correlated RNA and protein features into cross-omic modules, WGCNA provided a complementary view of coordinated variation that was not evident from individual-protein analyses. The Yellow module contained key extracellular matrix components (FN1, COL6A3, LAMB1, and POSTN) and protease-regulatory proteins (SERPINA3 and PZP), consistent with its functional enrichment profile [30,31,32,33,34,35,36,37,38,39,40]. ECM remodeling is relevant to both stromal alterations in high-risk lesions and early invasive growth.

Several snoRNAs highlighted in the present analysis have previously been implicated in cancer-associated processes, although evidence specific to breast cancer—and particularly to circulating snoRNAs—remains limited [22,23,27,41,42]. The recurrence of SNORD51, SNORD50B, SNORD38A, SNORD22, SNORD6, and SNORA73B [20,27,41,43,44,45,46,47,48] across differential, network, and external interaction analyses highlights these snoRNAs as candidates for further investigation. Most previous studies have examined tumor tissues or cancer cell lines, whereas our study extends this field by characterizing their abundance and cross-omic relationships in plasma. Given the distinct biological origins of tissue and circulating RNAs, the direction of change in plasma may not necessarily parallel that observed in tumor tissue.

Several Yellow-module proteins, including FN1, COL6A3, LAMB1, and POSTN, are established components of extracellular-matrix organization and remodeling, whereas SERPINA3 and PZP participate in protease regulation and circulating inflammatory or protein complexes [30,31,32,33,34,35,36,37,38,39,40]. Their coordinated occurrence with snoRNAs in the Yellow module provides a biologically coherent context for the matrix- and protease-related functions identified by enrichment analysis. Although none of these proteins was individually significant after multiple-testing correction, their collective module-level behavior highlights coordinated molecular relationships that would not have been evident from individual-protein analyses alone.

External contextualization using snoDB provided additional biological context for the exploratory plasma network [49]. Four of the 14 correlation-derived subnetwork edges (28.6%) corresponded to experimentally observed snoRNA interactions involving transcripts encoding the corresponding proteins. Furthermore, target-level enrichment was consistent with ECM organization and platelet activation/degranulation. Platelets are important reservoirs of circulating non-coding RNAs and growth factors and their activation may contribute to blood microparticle release during early tumor–stroma interactions [16]. The association of SNORD38A, SNORD22, SNORA73B, and SNORD6 with platelet-related processes suggests that circulating snoRNA dynamics may reflect systemic platelet responses associated with early breast cancer.

The CA group had a higher median age and BMI than the HB group and included two current smokers. These baseline differences may have influenced systemic RNA and protein profiles independently of disease status and should be considered when interpreting the observed molecular signatures.

Several study limitations must be acknowledged. First, the sample size was small (n = 32 total; n = 20 with paired multi-omic data), which limits the stability of WGCNA module topology and precludes complex multivariable adjustment for potential confounders such as age, BMI, or smoking status. Second, the cohort was restricted to White women, limiting generalizability to more diverse populations. Third, although snoDB provided experimentally observed interaction evidence, our data do not establish direct regulation, causality, or tissue origin. In addition, none of the individual RNA–protein correlations remained significant after multiple-testing correction, and the resulting subnetwork should therefore be considered hypothesis-generating. Furthermore, the FDR < 0.05 findings for SNORD51 and SNORD50B were restricted to the exploratory analysis of 25 Yellow-module RNA features. Because the Yellow module was identified from the same dataset, the module-restricted FDR adjustment does not account for the data-driven selection of module features and should not be interpreted as independent discovery-level significance. Finally, the plasma samples were obtained from a commercial biospecimen provider and prepared using a standardized single-centrifugation protocol. Although this approach provided consistent processing conditions across samples, residual platelet counts and platelet-specific quality-control data were unavailable. Therefore, contributions from residual platelets, platelet-derived extracellular vesicles, or ex vivo platelet activation to the observed platelet-associated molecular signatures cannot be distinguished from potential in vivo biological processes. The platelet-related functional enrichment should consequently be interpreted as exploratory and requires validation in independently collected samples with documented platelet-depletion procedures and residual platelet counts.

Future prospective studies in larger, multi-ethnic cohorts are warranted to validate candidate snoRNAs, including SNORD51, SNORD50B, SNORD38A, SNORA73B, using targeted RT-qPCR or digital PCR assays. In addition, fractionation of extracellular vesicles, platelets, and cell-free plasma will be essential to elucidate the release mechanisms, carrier states, and biological functions of circulating snoRNAs in early breast cancer.

In summary, integration of differential plasma snoRNA profiling, cross-omic network analysis, and experimentally observed snoRNA interaction data identified coordinated molecular patterns involving extracellular matrix/collagen and platelet/coagulation biology. These patterns and the associated candidate snoRNA–protein axes may characterize differences in the circulating molecular states of high-risk benign breast lesions and early breast cancer and warrant validation in independent cohorts.

## 4. Materials and Methods

### 4.1. Plasma Sample Preparation and Total RNA Isolation from Plasma

Thirty-two plasma samples from White female donors were obtained from the Corewell Health Biorepository (Grand Rapids, MI, USA) through Accio BiobankOnline (Suffolk, UK). The sample set comprised 9 CA, 14 HB, and 9 Be samples. All specimens were collected before surgery or systemic therapy. According to the provider-supplied protocol, blood was drawn into EDTA-containing Vacutainer tubes and transported immediately to the laboratory. Following centrifugation at 3500 rpm for 10 min at 23 °C, the plasma fraction was transferred into cryovials without disturbing the buffy coat. The cryovials were frozen overnight at −80 °C in a freezer puck, removed from the puck the following day, and maintained at −80 °C until use. Total RNA was isolated from 200 µL of plasma using the miRNeasy Serum/Plasma Kit (QIAGEN, Hilden, Germany) according to the manufacturer’s protocol. RNA was eluted in 14 µL of nuclease-free water and supplemented with 1 µL of RNase inhibitor. RNA quality and concentration were assessed using an Agilent 2100 Bioanalyzer (Agilent Technologies, Santa Clara, CA, USA).

### 4.2. Small RNA-Seq Library Preparation, Sequencing, and Bioinformatic Processing

Small RNA sequencing libraries were prepared by the Genomics and Bioinformatics Shared Resource (GBSR) at the University of Hawaii Cancer Center (UHCC). Library construction was performed using the QIAseq miRNA Library Kit and QIAseq miRNA NGS 12 Index IL (QIAGEN) according to the manufacturer’s instructions. Library quality was evaluated using an Agilent 2100 Bioanalyzer with a High Sensitivity DNA chip. The resulting libraries were sequenced as 76-bp single-end reads on an Illumina NextSeq 500 at the GBSR. Primary processing and UMI deduplication were conducted using the QIAGEN GeneGlobe Data Analysis Center. A total of 159 features were detected in the GeneGlobe other RNA category, comprising 31 SNORA, 105 SNORD, 13 SCARNA, and 10 other small non-coding RNA features. For the single-omic RNA analyses, 136 snoRNAs and 13 scaRNAs were retained, yielding a final set of 149 features.

### 4.3. Exploratory RNA Analysis and Differential Expression

The UMI count matrix and clinical metadata were imported into R, and sample identifiers were verified. Principal component analysis used variance-stabilizing transformed counts for all 149 RNA features across 32 samples; no abundance filter was applied for this analysis. Differential-expression analysis was performed with DESeq2 (version 1.52.0) [50] using a negative-binomial model. The pairwise comparisons were CA vs. HB, HB vs. Be, and CA vs. Be, with positive log2 fold change indicating higher abundance in the first group. Volcano plots highlighted nominal *p* < 0.05. Benjamini–Hochberg-adjusted *p* values were calculated [51], but the Figure 1B color classification was based on nominal *p* values to display exploratory differential-expression patterns consistently across the three pairwise comparisons. Overall expression differences among Be, HB, and CA were assessed using DESeq2 (version 1.52.0) LRT with a full model of ~Group and a reduced model of ~1. RNAs with nominal *p* < 0.05 were visualized using variance-stabilized expression values standardized by RNA (row Z-scores). RNAs were arranged according to their group-mean expression patterns to facilitate visualization, while samples were arranged by clinical group without assuming an ordered relationship among the three groups.

### 4.4. Data-Independent Acquisition (DIA) Proteomics

A grant from the IDeA National Quantitative Proteomics Resource supported DIA mass spectrometry analysis of 20 samples. Accordingly, 20 of the 32 plasma samples included in the RNA analysis were selected for proteomic analysis, comprising 9 CA and 11 HB samples. High-abundance plasma proteins were depleted using HighSelect Top14 Abundant Protein Depletion Resin (Thermo Fisher Scientific, Waltham, MA, USA). The remaining proteins were reduced, alkylated, and digested with sequencing-grade modified porcine trypsin (Promega, Madison, WI, USA) using S-Trap columns (ProtiFi, Fairport, NY, USA).

Tryptic peptides were separated on an XSelect CSH C18 column (2.5 µm, 150 mm × 0.075 mm; Waters, Milford, MA, USA) using an UltiMate 3000 RSLCnano system (Thermo Fisher Scientific) with a 60-min gradient from 2% to 35% buffer B. Buffers A and B contained 0.1% formic acid with 0.5% and 99.9% acetonitrile, respectively. Peptides were ionized by electrospray at 2.4 kV and analyzed on an Orbitrap Exploris 480 mass spectrometer (Thermo Fisher Scientific).

A chromatogram library was generated from six gas-phase fractions using 4 *m*/*z* DIA isolation windows at a resolution of 30,000. Wide-window acquisition consisted of a precursor scan from 385 to 1015 *m*/*z* at a resolution of 60,000, followed by 50 staggered DIA windows of 12 *m*/*z* at a resolution of 15,000. Optimized window placement was used for both acquisition schemes.

### 4.5. Proteomic Data Processing and Analysis

Proteomic data were processed by the IDeA National Resource for Quantitative Proteomics at the University of Arkansas for Medical Sciences. Peptides and proteins were identified and quantified against an empirically corrected spectral library using EncyclopeDIA [52] and visualized in Scaffold DIA, with false discovery rates controlled at 1% at both levels. Protein-exclusive MS2 intensities were evaluated using proteiNorm [53] and normalized by cyclic loess, yielding 422 quantified proteins.

Principal component analysis was performed using centered and scaled log_2_ intensities for the 394 proteins with complete measurements across all 20 samples. Differential abundance between 9 CA and 11 HB samples was evaluated for all 422 proteins using limma (version 3.68.5) with empirical Bayes moderation [54]. Log_2_ fold changes were calculated as CA relative to HB. *p* values were adjusted using the Benjamini–Hochberg method. Nominal *p* < 0.05 and FDR < 0.05 were evaluated, while volcano-plot colors were based on nominal *p* < 0.05. All proteomic analyses were considered exploratory.

### 4.6. Integrated snoRNA–Protein Network Analysis

Integrated network analysis was performed using the 20 samples with matched RNA and proteomic data. Features with measurements in at least 10 samples were retained, resulting in a 475-feature dataset comprising 422 proteins and 53 small non-coding RNAs. A signed network was constructed using WGCNA with a soft-thresholding power of 5 [55]. The adjacency matrix was converted to a topological overlap matrix, and modules were identified by hierarchical clustering and dynamic tree cutting with a minimum module size of 15. Module composition was summarized by molecular class.

### 4.7. Functional Enrichment and Yellow-Module Analysis

GO over-representation analysis was performed for the protein-coding genes corresponding to the proteins in each WGCNA module using enrichGO() in clusterProfiler (version 4.20.0) [56,57]. Biological Process, Molecular Function, and Cellular Component ontologies were evaluated, and *p* values were adjusted using the Benjamini–Hochberg method. For the Yellow module, protein-coding genes were ranked by CA vs. HB fold change and analyzed using gseGO() [56,57].

Differential-expression analysis of the 25 Yellow-module snoRNAs/scaRNAs was performed in the 20-sample subset using DESeq2 (version 1.52.0), with Benjamini–Hochberg correction across the 25 features. RNA counts were variance-stabilizing transformed for correlation analysis and compared with log_2_ cyclic-loess-normalized intensities for the 26 Yellow-module proteins. Spearman correlations were calculated for all 650 RNA–protein pairs. Differential-abundance strength, expressed as −log_10_(P), was also compared with each RNA’s mean correlation across the 26 proteins. The exploratory network retained positive RNA–protein correlations with nominal *p* < 0.05 connecting CA–HB-associated snoRNAs with proteins contributing to selected enriched GO terms [58].

### 4.8. External Functional Contextualization

Experimentally observed interactions for the seven snoRNAs represented in the exploratory network were retrieved from snoDB. Protein-coding transcript targets were compared with genes encoding the network proteins. GO Biological Process enrichment was performed using enrichGO() in clusterProfiler (version 4.20.0) [56], and Reactome pathway enrichment was performed using enrichPathway() in ReactomePA (version 1.56.0) [59,60]. Gene symbols were converted to Entrez identifiers using bitr() with org.Hs.eg.db (version 3.23.1). Enrichment analyses were conducted separately for the targets of each snoRNA, and *p* values were adjusted using the Benjamini–Hochberg method.

For the SNORD38A platelet-degranulation finding, 100 random target sets matched to the SNORD38A target-set size were sampled without replacement from the union of protein-coding targets of the seven snoRNAs and analyzed using the same Reactome enrichment procedure. The empirical *p* value was calculated as (b + 1)/(B + 1), where b was the number of random sets with an adjusted *p* value equal to or smaller than that observed for SNORD38A and B = 100.

### 4.9. Statistical Analysis

Continuous variables are reported as medians and interquartile ranges, and categorical variables as numbers and percentages. All tests were two-sided. Nominal *p* < 0.05 was used for exploratory comparisons, and Benjamini–Hochberg-adjusted *p* values were reported for multiple-testing analyses. Because of the limited sample size, all differential-abundance, network, and correlation analyses were considered exploratory.

## Figures and Tables

**Table 1:** Demographic characteristics of the plasma samples.

Characteristic	Be (n = 9)	HB (n = 14)	CA (n = 9)
Age at plasma collection, years, median (IQR)	54 (50–63)	51 (49–64)	61 (47–64)
BMI, kg/m^2^, median (IQR)	26 (23–30)	25 (23–30)	30 (29–32)
**Smoking Status, n (%)**			
Never	3 (37.5%)	10 (76.9%)	1 (11.1%)
Former	5 (62.5%)	3 (23.1%)	6 (66.7%)
Current	0 (0%)	0 (0%)	2 (22.2%)
Missing	1	1	0

Data are presented as median (IQR) or n (%). Percentages for smoking status were calculated among samples with available data. BMI data were available for 6 Be, 13 HB, and 9 CA samples. Smoking status was available for 8 Be, 13 HB, and 9 CA samples. All samples were from White women.

**Table 2:** Molecular composition of the four selected WGCNA modules.

Module	snoRNA/scaRNA	Other Small ncRNA	Protein
Yellow	SNORA3B, SNORA73B, SNORD102, SNORD103C, SNORD104, SNORD119, SNORD20, SNORD22, SNORD24, SNORD25, SNORD26, SNORD31, SNORD38A, SNORD38B, SNORD3A, SNORD50B, SNORD51, SNORD6, SNORD63, SNORD66, SNORD74, SNORD87 (U87), SNORD95, SCARNA6, SCARNA9	RNU1-1	FN1, CP, A2M, PZP, C7, TLN1, SERPINA3, HRG, COL6A3, SHBG, LRP1, TUBB1, IGFBP2, VNN1, H6PD, NCAM1, LAMB1, FCGR3B, NAGLU, LYVE1, POSTN, IGF2R, IGKV1-8, ITLN1, SH3BGRL3, CADM1
Brown		RNU12, RNY1	ITIH1, THBS1, MST1, CPN2, HBB, CAT, SLC4A1, CPB2, HBD, PRDX2, CPN1, CA1, C4BPB, SPTB, ANK1, MBL2, HBA1, IGKC, C1QC, GAPDH, PPBP, CA2, LDHB, PF4, SELL, IL1RAP, CST3, PRDX6, PPIA, EPB42, FCGR3A, TPI1, SELENBP1, BLVRB, BPGM, APOC2, IGHV3-49, S100A8, PRDX1, CSF1R, ICAM2, SPARC, LILRA2, HBG1, HBG2, MDH1, ZYX, PRG2, STOM, KRT71, KRT74, KRT72, KRT73
Purple	SNORD89		TFRC, IGHA1, IGA2, TGFBI, SAA1, IGKV1-6, IGHV4-61, IGHV4-30-4, IGHV4-31, IGHV4-38-2, IGHV4-39, IGHV4-59, IGHV4-34, IGHV3-43D, IGHV3-9, CD44
Green	SNORA33, SNORA36A, SNORA36B, SNORA51, SCARNA15, SCARNA3, SCARNA8	RNY5, Ro-associated	C4B, FLNA, TF, APOA4, KNG1, PROS1, GPLD1, VCL, HPR, ACTB, ACTG1, ACTC1, ITGA2B, PCYOX1, HSPA8, APOC3, ITGB3, FERMT3, CFHR3, APOC4, TAGLN2, YWHAZ, MYL12B, MYL12A, FAH, MYL6, RAP1B, PFN1, PKM, ACTBL2, PLEK, MARCO, GSTO1, CFL1, CAP1, CORO1A, YWHAG, WDR1

WGCNA, weighted gene co-expression network analysis; snoRNA, small nucleolar RNA; scaRNA, small Cajal body-specific RNA.

## Data Availability

The small RNA sequencing data analyzed in this study are publicly available in the NCBI Gene Expression Omnibus (GEO) under accession number GSE225117.

## Supplementary Materials

The following supporting information can be downloaded at https://www.mdpi.com/article/10.3390/ijms27219530/s1.
