# Supplementary material for: Integrated Plasma snoRNA–Protein Network Analysis Identifies Circulating Molecular Signatures Associated with High-Risk Benign Breast Lesions and Early Breast Cancer
Source: Int J Mol Sci. 2026 Oct 25;27(21):9530. doi: 10.3390/ijms27219530 (PMC13763387; doi:10.3390/ijms27219530)
Supplement: Supplementary file 1 [file ijms-27-09530-s001.zip › Supplementary Table S1. Clinicopathological information of the plasma samples_mj.pdf]

Supplementary Table S1. Clinicopathological information of the plasma samples

Group	Case ID	Sex	Race	BMI	Smoking Status	Age at Plasma Acquisition	Histologic Type (First letter Capitalize)	Notes
High-risk benign	HB1	F	White	34	Never	51	ADH	
	HB2	F	White	23	Never	67	ADH	
	HB3	F	White	24	Former	70	ADH involving papilloma	
	HB4	F	White	29	Former	61	ALH	
	HB5	F	White	33	Never	65	ALH and papilloma	ER (+)
	HB6	F	White	25	Never	51	FEA, fibrocystic changes, usual ductal hyperplasia	
	HB7	F	White	21	Never	45	FEA, focal microcalcifications, fibrocystic change, focal usual ductal hyperplasia	
	HB8	F	White	31	Former	61	Focal ALH	
	HB9	F	White	22	Never	73	Focal ALH and focal ductal hyperplasia with microcalcifications	
	HB10	F	White	23	Never	50	Focal FEA	
	HB11	F	White	19	Never	51	Focal FEA, focal radial scar, usual ductal hyperplasia	
	HB12	F	White	30	Never	49	ADH	
	HB13	F	White	26	Never	48	Multifocal FEA	
	HB14	F	White	N/A	N/A	47	Right breast ADH, Left breast ALH	
Benign	Be1	F	White	20	Never	52	Benign breast tissue, stromal fibrosis, small fibroadenomas	BRCA2 positive
	Be2	F	White	30	Former	63	Benign breast tissue, focal fibrocystic changes	
	Be3	F	White	29	Former	42	Fibroadenoma	
	Be4	F	White	N/A	Former	50	Fibroadenoma	
	Be5	F	White	N/A	Former	54	Fibroadenoma	
	Be6	F	White	N/A	N/A	63	Fibroadenoma	BRCA2 positive
	Be7	F	White	23	Never	41	Fibrocystic changes, fibroadenomatiod mastopathy, focal microcalcifications	
	Be8	F	White	31	Never	65	fibrocystic change, microcalcifications	
	Be9	F	White	23	Former	58	Microcalcifications, fibrocystic changes, benign ducts and lobules	

Group	Case ID	Sex	Race	BMI	Smoking Status	Age at Plasma Acquisition	Histologic Type	pTNM Stage	Histologic Grade	ER	PR	Her2 (IHC)	Subtype
Early Stage Cancer	CA1	F	White	30	Current	42	IDC, NOS	pT1a/pN0/pMX	Grade 1	Positive	Positive	Negative	LA
	CA2	F	White	29	Former	62	IDC, NOS	pT1a/pN0/pMX	Grade 1	Positive	Positive	Negative	LA
	CA3	F	White	38	Former	47	Invasive papillary carcinoma	pT1a/pN0(sn)/pMX	Grade 1	Positive	Positive	Negative (1+)	LA
	subCA4	F	White	32	Never	67	IDC with metaplastic features	pT1b/pN0/pMX	Grade 3	Negative	Negative	Negative	TN
	CA5	F	White	33	Current	40	Metaplastic carcinoma, spindle cell type	pT1a/pN0/pMX	Grade 3	Negative	Negative	Negative	TN
	CA6	F	White	30	Former	66	IDC, NOS	pT1b/pN0(sn)/pMX	Grade 1	Positive	Positive	Negative	LA
	CA7	F	White	29	Former	64	IDC, NOS	pT1b/pN0(sn)/pMX	Grade 3	Negative	Negative	Negative	TN
	CA8	F	White	18	Former	55	ILC	pT1b/pN0/pMX	Grade 1	Positive	Positive	Negative	LA
	CA9	F	White	21	Former	61	DCIS	*ypTis/ypN0/ypMX	N/A	Negative	Negative	Negative	TN

Abbreviations: ADH, atypical ductal hyperplasia; ALH, atypical lobular hyperplasia; FEA, flat epithelial atypia; IDC, invasive ductal carcinoma; NOS, not otherwise specified; ILC, invasive lobular carcinoma; DCIS, ductal carcinoma in situ; sn, sentinel node; N/A, not available; LA, luminal A; TN, triple-negative. *: CA9 received neoadjuvant therapy after blood collection but before surgery.

[grey box] : Samples used for paired snoRNA-Protein analysis
